# Complications and management of ventriculoatrial (VA) shunts, a case report

**DOI:** 10.1186/1743-8454-7-S1-S42

**Published:** 2010-12-15

**Authors:** David L Nash, Kevin Schmidt

**Affiliations:** 1 Department of Physical Medicine and Rehabilitation Mayo Clinic 200 First Street SW Rochester, MN 55905, USA

## Background

An 18 year old female with history of myelomeningocele and hydrocephalus had her ventriculoperitoneal shunt converted to a VA shunt following development of peritonitis at age 11 years. Seven years later she was admitted with abdominal pain. CT revealed a perihepatic abscess and incidental right lower lobe pulmonary embolus (PE). Further investigation revealed a large right cardiac ventricle thrombus. She underwent open thrombectomy and was anticoagulated. Pathologic evaluation of the thrombus demonstrated focal purulent inflammation without identifiable organisms.

## Materials and methods

A literature review of VA shunt complications and long-term management was performed. Paediatric infectious disease, cardiology and neurosurgery were consulted. Long term anticoagulation with warfarin was instituted.

## Results

Peri-procedural antibiotic prophylaxis was not recommended. Neurosurgery plans to reevaluate and possibly convert the shunt to a ventriculopleural shunt in the future.

## Conclusions

There is a paucity of literature regarding the complications and long term management of VA shunts. Reviews suggest the rate of clinically evident PE and pulmonary hypertension in patients with VA shunts is only 0.4 and 0.3 percent respectively. Autopsy studies however have demonstrated rates as high as 59.7 and 6.3 percent. Progressive cor pulmonale occurs in a small percentage of patients with VA shunts, but carries a high mortality rate. The contribution of subclinical infection or occult thrombophilia in subsequent thromboembolic disease is unknown. This begs the following questions. 1. Should all patients with VA shunts be anticoagulated, to what extent? 2. Is there a role for thrombophilia screening to detect at risk subjects? 3. Does prophylactic antibiotic use have a role? 4. What type of surveillance for asymptomatic PE and pulmonary hypertension is prudent? 5. Is surgical conversion of VA shunts recommended? Long-term anticoagulation is a difficult recommendation to generalize to an asymptomatic population of patients with risks ranging from medication interactions to hemorrhage. Given the prevalence of subclinical thromboembolism however, pharmacologic anticoagulation remains a strong consideration that must be individualized. Currently the American Heart Association finds no convincing evidence that peri-procedural prophylaxis is indicated in VA shunt patients. Finally, surgical conversion of VA shunts may be considered in efforts to reduce the potential for life-threatening complications of VA shunts. In conclusion, clinically evident and subclinical thromboembolic disease is a known complication of VA shunts. Management and surveillance of these patients is largely undefined in the current literature. Long term anticoagulation, thrombophilia screening, vigilant follow up, and surgical revision remain considerations.

